# Mapping of QTL for Grain Yield Components Based on a DH Population in Maize

**DOI:** 10.1038/s41598-020-63960-2

**Published:** 2020-04-27

**Authors:** Jiwei Yang, Zonghua Liu, Qiong Chen, Yanzhi Qu, Jihua Tang, Thomas Lübberstedt, Haochuan Li

**Affiliations:** 1 Agronomy College of Henan Agricultural University/Key Laboratory of Wheat and Maize Crops Science/ Collaborative Innovation Centre of Henan Grain Crops, Zhengzhou, 450002 China; 2 Department of Agronomy, Iowa State University, Ames, IA 50011 USA

**Keywords:** Plant genetics, Plant genetics, Plant breeding, Plant breeding

## Abstract

The elite maize hybrid Zhengdan 958 (ZD958), which has high and stable yield and extensive adaptability, is widely grown in China. To elucidate the genetic basis of yield and its related traits in this elite hybrid, a set of doubled haploid (DH) lines derived from ZD958 were evaluated in four different environments at two locations over two years, and a total of 49 quantitative trait loci (QTL) and 24 pairs of epistatic interactions related to yield and yield components were detected. Furthermore, 21 QTL for six investigated phenotypic traits were detected across two different sites. Combining the results of these QTL in each environment and across both sites, three main QTL hotspots were found in chromosomal bins 2.02, 2.05–2.06, and 6.05 between the simple sequence repeat (SSR) markers umc1165-bnlg1017, umc1065-umc1637, and nc012-bnlg345, respectively. The existence of three QTL hotspots associated with various traits across multiple environments could be explained by pleiotropic QTL or multiple tightly linked QTL. These genetic regions could provide targets for genetic improvement, fine mapping, and marker-assisted selection in future studies.

## Introduction

Maize is one of the most important food and feed crops in the world and plays an important role in ensuring food security. In maize breeding, increasing grain yield is the primary objective. Because grain yield is a complex quantitative trait controlled by multiple quantitative trait loci (QTL) with small effects that are affected by significant genotype-by-environment (G × E) interactions^1–3^, the heritability of grain yield is usually lower than those of other traits, such as plant height, ear height, ear length (EL), ear row number (ERN), and 100 kernel weight (HKW)^4–7^. A low heritability indicates that a trait is affected by not only genotype but also G × E interactions^8^. Thus, multi-environment trials are necessary to reach reliable conclusions. In previous studies, EL, ERN, HKW, ear diameter (ED), kernel number per row (KNR), kernel percentage (KP), and kernel weight per ear (KWE) were proven to be important yield components in maize, and these traits were positively correlated with grain yield^6,9–11^. Yield components usually have higher heritabilities; for example, in the study of Flint-Garcia *et al*.^8^, the heritabilities of ERN and EL were 90.5% and 87.2%, respectively, whereas the heritability of grain yield was only 83.7%. In another study, the heritability of grain yield (81%) was also lower than those of its components KNR, EL, and HKW (91%, 86%, and 84%, respectively)^12^. Ma *et al*.^5^ also found that grain yield had the lowest broad-sense heritability of 77.4% compared to its three components ERN (88.2%), EL (84.6%), and HKW (84.9%). Thus, selection for yield components could be more effective than direct selection for grain yield itself.

QTL mapping has been widely used to detect the genetic basis underlying yield components in maize^4,9,13–16^, and multiple QTL for grain yield components have been detected, but most of them made minor contributions to yield. Lima *et al*.^7^ identified 16 QTL related to grain yield in five environments using a set of 256 F_2:3_ families derived from tropical maize germplasm. Yan *et al*.^17^ detected 29 QTL for grain yield and yield components at two locations using an F_2:3_ population. In another study, 12 major QTL for grain weight per plant (GWP) and HKW were identified using two F_2:3_ populations across six environments, and the highest contribution of a single QTL was 8%^4^. Zhou *et al*.^18^ developed a population with a narrow genetic base from the cross of Ye478 and its chromosome segment substitution line SL17-1 in maize and detected one major QTL responsible for EL, KNR, and GWP in chromosomal bin 7.02. In a recent study, 26 QTL and 6 SNPs associated with ear and grain morphological traits were detected across four environments by using a genome-wide association analysis (GWAS)^11^.

In contrast to the F_2:3_ or backcross populations used for QTL mapping, recombinant inbred line (RIL) and doubled haploid (DH) line populations consist of genetically stable families and can be used to obtain more accurate and effective phenotyping for QTL mapping. RIL populations are usually developed by continuous self-pollination for more than eight generations, which is a time-consuming and expensive process. In contrast, DH populations are produced in only two generations. Thus, DH populations are increasingly used for mapping experiments in various species^19–24^.

The maize hybrid Zhengdan 958 (ZD958), a commercial hybrid with high and stable yield, was widely grown on approximately 500 million hectares between 2001 and 2015 in China^25^ and is still a competitive variety in the northern and central parts of China. Because its two parent lines, Zheng58 (Z58) and Chang7-2 (C7-2) have high general combining ability and represent the two main heterotic groups in China, Reid and Tangsipingtou, this hybrid and its parental lines have been intensively studied for heterosis^26^, cultivation conditions and the physiological basis of high yield^27,28^. However, the genetic basis of the high yield of the elite hybrid ZD958 and its components remain little known. In this study, a set of DH populations of ZD958 was developed and evaluated in four different environments at two locations across two years (2014 and 2015). The objectives of this study were to (1) elucidate the relationship between grain yield and its components, (2) identify QTL for grain yield-related traits across multiple environments, and (3) study G × E interactions. These findings may reveal the genetic basis of grain yield and its components in the hybrid ZD958 and provide molecular markers for developing new superior maize hybrids.

## Results

### Phenotypic performance in different environments

There were 161 DH families obtained from the hybrid ZD958 by *in vivo* haploid induction, haploid identification, and haploid genome doubling. The DH population, the hybrid ZD958 and its two parental lines were evaluated in four different environments at two locations over two years (Table 1). For the two parents, the EL, HKW, and GWP of the inbred line Z58 were higher than those of C7-2, and the other traits (ED, ERN, and KNR) were lower. For the hybrid ZD958, all six estimated traits were higher than those of the two parent lines (except for ERN at the Changge location in 2015). For the DH population, the mean values of all the traits were between the values of the two parents and lower than those of the hybrid ZD958. Additionally, the traits of the DH population followed a normal distribution with kurtosis and skewness <1 (Table 1, Supplementary Fig. S1), and the average coefficient of variation (CV) was highest for GWP at 24.7%, while that of ED was lowest with only 6.8%.

**Table 1:** Phenotypes of grain yield components in DH families and parental lines.

Traits	Loc.	Z58	C7-2	ZD958	DH population			
					Mean ± sd	Kurtosis	Skew	CV (%)
EL (cm)	CG14	11.98	8.64	16.31	9.87 ± 1.56	−0.32	0.27	15.82
	QX14	13.94	10.85	17.24	11.7 ± 1.8	−0.44	−0.06	15.36
	CG15	11.94	9.17	16.51	10.5 ± 1.76	−0.37	−0.34	16.75
	QX15	15.19	11.03	18.51	11.77 ± 1.87	−0.21	0.06	15.86
	Mean	13.26	9.92	17.14	10.97 ± 1.56	−0.51	−0.08	14.23
ED (cm)	CG14	3.53	4.4	4.61	3.45 ± 0.31	0.33	−0.3	9.03
	QX14	3.73	4.38	5.03	3.86 ± 0.3	0.63	−0.2	7.75
	CG15	3.42	3.66	4.42	3.48 ± 0.29	0.36	0.1	8.3
	QX15	4.09	4.24	5.26	3.83 ± 0.32	−0.17	0.21	8.36
	Mean	3.69	4.17	4.83	3.66 ± 0.25	−0.49	−0.08	6.77
ERN	CG14	12.5	14.4	14.8	12.88 ± 2	0.94	0.85	15.54
	QX14	11.4	16	16.42	13.54 ± 2.02	0.16	0.43	14.94
	CG15	11.4	14.8	14.4	12.89 ± 1.82	0.5	0.67	14.13
	QX15	12	14.33	14.85	12.94 ± 1.68	−0.16	0.09	13.01
	Mean	11.83	14.88	15.12	13.04 ± 1.73	0.06	0.55	13.27
KNR	CG14	17.2	19.75	38.7	19.04 ± 3.94	0.15	−0.16	20.68
	QX14	20.6	25.5	40.18	21.82 ± 4.38	−0.36	−0.04	20.09
	CG15	17.8	20.3	36.7	19.06 ± 3.64	−0.1	0.04	19.1
	QX15	20.43	23.08	40.43	20.81 ± 4.09	−0.09	−0.12	19.63
	Mean	19.01	22.16	39	20.15 ± 3.48	−0.08	0.07	17.28
HKW (g)	CG14	21.55	14.99	26.51	17.71 ± 3.25	−0.51	0.01	18.36
	QX14	26.68	22.6	41.57	25.6 ± 4.24	−0.48	0.04	16.54
	CG15	20.35	14.6	23.4	18.57 ± 4.07	0.45	0.58	21.9
	QX15	35.8	27.45	42.4	28.72 ± 4.28	−0.6	0.01	14.92
	Mean	26.1	19.91	33.47	22.73 ± 3.48	−0.45	0.14	15.53
GWP (g)	CG14	39.08	35.36	140.45	35.8 ± 10.61	0.59	0.57	29.12
	QX14	78.15	61.46	234.18	62.29 ± 18.49	−0.32	−0.02	29.68
	CG15	39.78	35.53	124.31	38.43 ± 11.87	−0.36	0.2	30.89
	QX15	78.04	65.98	245.44	68.33 ± 19.53	−0.05	0.32	28.58
	Mean	58.76	49.58	186.1	51.29 ± 12.68	0.14	0.18	24.72

Note: EL, ear length; ED, ear diameter; ERN, ear row number; KNR, kernel number per row; HKW, hundred kernel weight; GWP, grain weight per plant; sd, standard deviation; CV, coefficient of variation.

### Variance and correlation analysis

Highly significant variations (P = 0.01) were found between genotypes, environments and G × E interactions for the investigated traits (Table 2), and no significant variation was found between replications. In the correlation analysis, GWP was significantly positively correlated with the other traits (Table 3), indicating that EL, ED, ERN, KNR, and HKW could contribute to increasing GWP. The correlation coefficients were lowest (0.21–0.39) between HKW and GWP in the four environments. However, HKW was not significantly correlated with EL and ED. There was a significant (P = 0.01) negative correlation between HKW and ERN in the four environments and with KNR at the Changge location in 2014 and 2015. Additionally, GWP was closely and positively correlated with KNR (r^2^ = 0.66 to 0.85) in different environments. In contrast to the heritability of ERN (94.5%) and EL (90.9%), the heritability of the other traits (ED, KNR, HKW, and GWP) was lower, but the lowest value of *H*^2^, which was for ED, still exceeded 82% (Table 2).

**Table 2:** ANOVA for ear traits for the DH population in four environments.

Source of variation	EL	ED	ERN	KNR	HKW	GWP
Rep.	0.069	0.091	0.001	0.1	0.91	0.001
Env.	224.48**	233.68**	31.38**	93.89**	1529.36**	793.06**
Gen.	17.38**	7.98**	27.07**	15.68**	17.29**	12.22**
Env.× Gen.	1.59**	1.40**	1.31**	1.66**	2.15**	2.17**
H^2^ (%)	90.88	82.48	94.54	89.81	88.33	83.82

Note: **significance at the 0.01 level; Rep, replication; Env, environments; Gen., genotype; H^2^_,_ heritability.

**Table 3:** Pairwise correlation coefficients between the six ear traits across four environments.

Year	Trait	EL	ED	ERN	KNR	HKW	GWP
2014	EL		0.25**	0.27**	0.64**	−0.01	0.56**
	ED	0.29**		0.6**	0.43**	0.14	0.62**
	ERN	0.22**	0.64**		0.41**	−0.32**	0.48**
	KNR	0.67**	0.41**	0.35**		−0.09	0.81**
	HKW	0.02	0.15	−0.36**	−0.22**		0.21**
	GWP	0.56**	0.55**	0.33**	0.66**	0.33**	
2015	EL		0.37**	0.35**	0.64**	0.02	0.63**
	ED	0.35**		0.7**	0.55**	0.14	0.73**
	ERN	0.25**	0.6**		0.5**	−0.25**	0.56**
	KNR	0.64**	0.46**	0.37**		−0.03	0.85**
	HKW	0.06	0.14	−0.4**	−0.19*		0.26**
	GWP	0.52**	0.64**	0.29**	0.7**	0.39**	

Note: * and ** indicate significance levels of P < 0.05 and P < 0.01, respectively; correlation coefficients for CG14 are below the diagonal, while those for QX14 are above the diagonal; correlation coefficients for CG15 are below the diagonal, while those for QX15 are above the diagonal.

### Genetic map construction

A total of 119 polymorphic simple sequence repeat (SSR) primer pairs were identified from 897 markers on ten chromosomes in MaizeGDB (http://www.maizegdb.org/) and used to construct linkage maps for QTL detection. The linkage map covered all 10 maize chromosomes with a total genome size of 2315 cM, the average size of the marker intervals was 19 cM, and all the marker positions were consistent with the linkage map for maize B73 × Mo17 (IBM) (http://www.maizegdb.org). DH populations can be obtained by tissue culture and *in vivo* haploid induction; the former is usually affected by maternal genotype and is prone to segregation distortion, while the latter is more likely to be consistent with Mendelian inheritance. In the present study, the mapping population was a DH population developed by *in vivo* haploid induction rather than tissue culture, and none of the molecular markers used for linkage mapping showed significant segregation distortion.

### QTL detection in single environments

**Figure 1:**
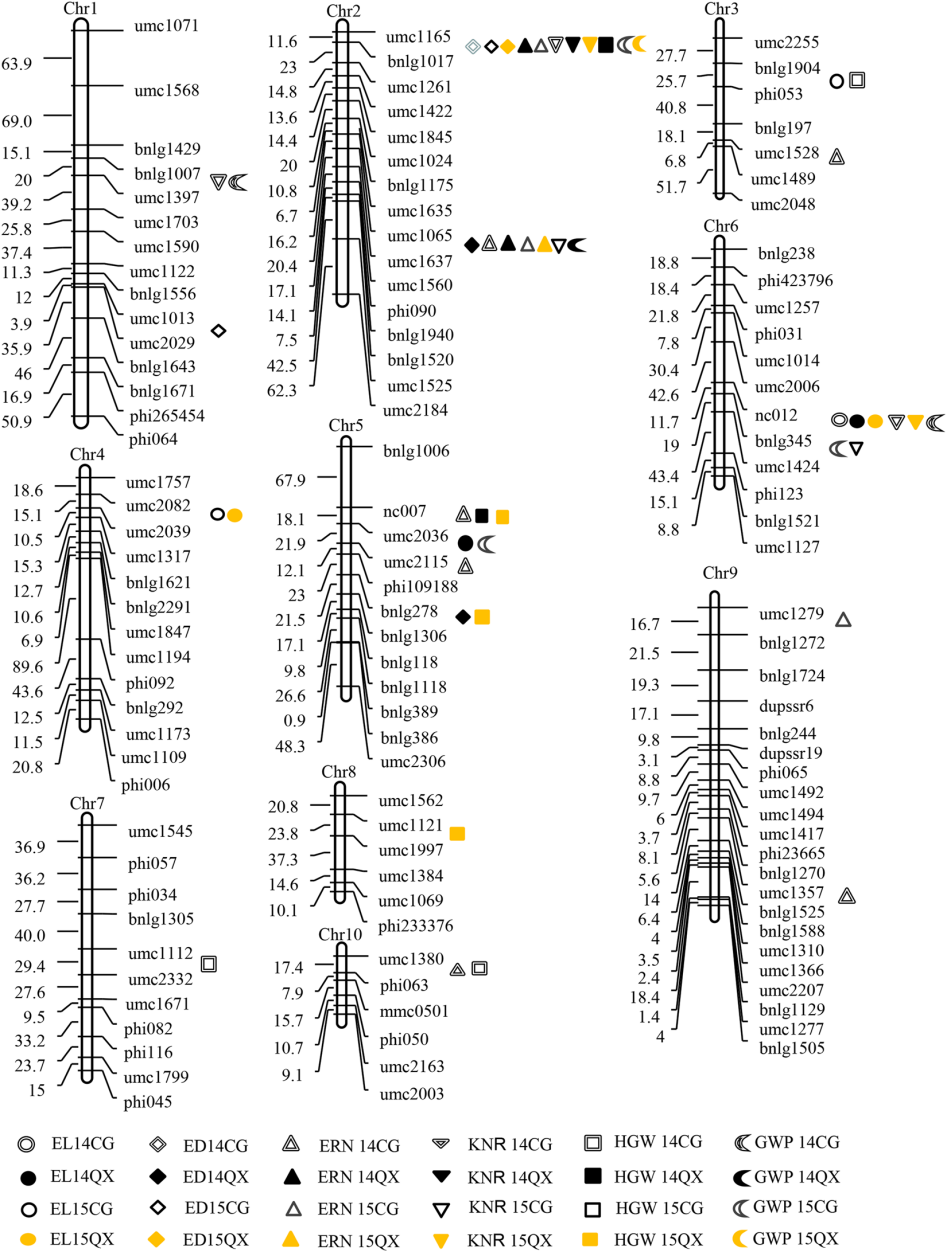
Molecular linkage map of DH families and distribution of QTL for ear traits in four environments over 2 years. Note: The numbers on the left side of each chromosome represent the genetic distances between the two flanking markers in centiMorgans (cM). The right side of each chromosome shows the polymorphic markers. The different shapes beside the markers represent different QTL; the circle, diamond, triangle, inverted triangle, square and crescent shapes represent ear length (EL), ear diameter (ED), ear row number (ERN), kernel number per row (KNR), 100-kernel weight (HKW), and grain weight per plant (GWP), respectively. Double line graphics, black graphics, white graphics, and yellow graphics represent the corresponding traits detected at Changge in 2014 (CG14), Qixian in 2014 (QX14), Changge in 2015 (CG15) and Qixian in 2015 (QX15), respectively.

In this study, a total of 49 QTL were detected (Table 4); 17, 10, 12, and 10 QTL were identified for the traits measured at Changge in 2014 (CG14), Qixian in 2014 (QX14), Changge in 2015 (CG15) and Qixian in 2015 (QX15), respectively. These QTL were distributed on 10 chromosomes, and most QTL were located on chromosomes 2, 5, and 6, which had 18, 8, and 9 QTL, respectively (Fig. 1). Each QTL explained a percentage of phenotypic contribution from 5.7 to 17.8%, including 20 main effect QTL with more than 10% contribution and 4 QTL with contributions over 15%. Five and three QTL from these were detected across two and three environments. However, only one QTL was detected in all four environments (Table 4, Fig. 1); it was on chromosome 2, and it explained 8.5, 11,5, 9.4 and 11.0% of the phenotypic variation in CG14, QX14, CG15, and QX15, respectively. These loci originated from the inbred parental line C7-2, which contained favourable alleles controlling ERN.

**Table 4:** Single-environment QTL identified for ear traits in the DH population in each environment.

Trait	QTL	Marker interval	Bin	Env.	LOD	R^2^ (%)	A
EL	*qEL3*	bnlg1904-phi053	3.04	CG15	4.14	13.12	−0.66
	*qEL4*	umc2082-umc2039	4.03	CG15	3.52	10.43	−0.61
				QX15	3.18	9.00	−0.57
	*qEL5*	umc2306-bnlg1306	5.06–5.07	QX14	5.01	12.51	−0.65
	*qEL6a*	phi031-umc1014	6.04	CG14	2.96	7.69	−0.45
	*qEL6b*	nc012-bnlg345	6.05	CG14	5.24	12.28	0.58
				QX14	2.83	9.19	0.56
				QX15	4.11	10.04	0.61
ED	*qED1*	umc1013-umc2029	1.08	CG15	2.60	6.21	−0.09
	*qED2a*	umc1165-bnlg1017	2.02	CG14	3.34	11.19	0.11
				CG15	3.21	9.60	0.10
				QX15	2.51	6.24	0.09
	*qED2b*	umc1065-umc1637	2.05–2.06	QX14	2.95	7.59	0.08
	*qED5*	bnlg278-bnlg1306	5.04	QX14	2.88	9.86	−0.10
ERN	*qERN2a*	umc1165-bnlg1017	2.02	QX14	3.00	6.80	0.58
				CG15	3.00	7.62	0.55
	*qERN2b*	umc1065-umc1637	2.05–2.06	CG14	3.95	8.47	0.58
				QX14	4.11	11.50	0.68
				CG15	3.20	9.42	0.55
				QX15	3.40	10.99	0.56
	*qERN3*	umc1528-umc1489	3.07	CG14	3.70	7.88	−0.55
	*qERN5a*	nc007-umc2036	5.01	CG14	5.50	17.55	0.87
	*qERN5b*	umc2115-phi109188	5.02	CG14	3.77	9.78	−0.67
	*qERN9a*	umc1357-bnlg1505	9.05	CG14	2.68	7.41	−0.60
	*qERN9b*	umc1279-bnlg1272	9.0	CG15	2.56	6.63	0.51
	*qERN10*	umc1380-phi063	10.02	CG14	3.2	9.97	0.64
KNR	*qKNR1*	bnlg1007-umc1397	1.03	CG14	2.72	11.00	1.36
	*qKNR2a*	umc1165-bnlg1017	2.02	CG14	2.59	9.44	1.29
				QX14	3.20	11.16	1.60
				QX15	4.95	14.86	1.72
	*qKNR2b*	umc1065-umc1637	2.05–2.06	CG15	3.84	8.72	1.11
	*qKNR6a*	nc012-bnlg345	6.05	CG14	2.60	8.54	1.19
				QX15	2.54	5.92	1.04
	*qKNR6b*	bnlg345-umc1424	6.05	CG15	4.61	17.7	1.59
HKW	*qHKW2*	umc1165-bnlg1017	2.02	QX14	4.65	10.44	−1.52
	*qHKW3*	bnlg1904-phi053	3.05	CG14	3.72	8.26	0.98
	*qHKW5a*	nc007-umc2036	5.01	QX14	2.74	7.07	−1.15
				QX15	3.39	11.63	−1.50
	*qHKW5b*	bnlg278-bnlg1306	5.07	QX15	3.77	8.28	−1.34
	*qHKW7*	umc1112-umc2332	7.03	CG14	3.24	14.73	−1.28
	*qHKW8*	umc1121-umc1997	8.05	QX15	2.67	5.71	−1.05
	*qHKW10*	umc1380-phi063	10	CG14	2.63	6.54	−0.86
GWP	*qGWP1*	bnlg1007-umc1397	1.02	CG14	4.50	16.94	4.40
	*qGWP2a*	umc1165-bnlg1017	2.02	CG15	2.71	6.85	3.46
				QX15	2.71	6.54	5.67
	*qGWP2b*	umc1065-umc1637	2.05–2.06	QX14	3.51	10.61	6.11
	*qGWP5*	umc2036-umc2115	5.01	CG15	3.49	11.33	−4.07
	*qGWP6a*	nc012-bnlg345	6.05	CG14	3.20	9.04	3.25
	*qGWP6b*	bnlg345-umc1424	6.06	CG15	4.20	17.84	5.26

Note: LOD, logarithm of odds for each QTL; R^2^, contribution rate; A, additive effect of the QTL. Negative values indicate that the alleles for increased trait value were contributed by the parent Zheng58; positive values indicate that the alleles for increased trait value were contributed by the other parent, Chang7–2.

### QTL detected in different locations

The two locations, Changge (CG) and Qixian (QX), are located in the central and northern parts of Henan Province, respectively, and belong to two important maize planting zones in China. A total of 21 QTL for six phenotypic traits were detected across these two locations (Table 5), and 11 and 10 QTL were identified in CG and QX, respectively. There were ten QTL located on chromosome 2; three QTL on chromosomes 3, 5, and 6; and only one QTL on chromosomes 9 and 10 (Supplementary Fig. S2). Each QTL explained a percentage of phenotypic variation from 6.0 to 18.8%, including nine main effect QTL with more than 10% contribution to variation. Five consistent QTL were simultaneously detected in both locations, of which 2, 1, 1, and 1 QTL were identified for EL, ERN, HKW, and GWP on chromosomes 2, 3, 5, and 6, respectively. The QTL *qEL3-Z* on chromosome 3 showed the largest contribution, with a value of 18.8% at the QX site. Notably, *qERN*2*-Z* and *qGWP*2*a-Z* were in the same marker interval, umc1065-umc1637. QTL for ED and KNR were also identified in this region at the CG and QX planting sites, respectively. These results suggest a close genetic correlation among ED, ERN, KNR, and GWP and could be due to pleiotropy of this QTL.

**Table 5:** QTL identified for ear traits in the DH population in each planting zone.

Trait	QTL	Marker interval	Bin	Location	LOD	R^2^ (%)	A
EL	*qEL3-Z*	bnlg1904-phi053	3.04	CG	2.67	9.31	−0.52
				QX	5.49	18.8	−0.71
	*qEL6-Z*	nc012-bnlg345	6.05	CG	2.65	6.21	0.43
				QX	3.31	8.87	0.48
ED	*qED2a-Z*	umc1422-umc1845	2.03	QX	2.97	10.05	0.08
	*qED2b-Z*	umc1065-umc1637	2.05–2.06	CG	4.04	11.18	0.09
	*qED5-Z*	bnlg278-bnlg1306	5.04	CG	2.76	9.61	−0.1
ERN	*qERN2-Z*	umc1065-umc1637	2.05–2.06	CG	4.75	13.71	0.65
				QX	4.17	11.29	0.6
	*qERN3-Z*	umc1528-umc1489	3.07	QX	3.12	7.85	−0.51
	*qERN9-Z*	umc1279-bnlg1272	9.00	CG	3.5	7.44	0.5
KNR	*qKNR2a-Z*	umc1165-bnlg1017	2.02	CG	5.84	17.26	1.73
	*qKNR2b-Z*	umc1065-umc1637	2.05–2.06	QX	3.05	7.95	1.09
	*qKNR10-Z*	phi063-mmc0501	10.02	QX	2.62	6.73	1
HKW	*qHKW2-Z*	umc1165-bnlg1017	2.02	CG	4.13	8.78	−1.3
	*qHKW5-Z*	nc007-umc2036	5.01	CG	5.4	14.64	−1.52
				QX	4.5	13.82	−1.46
GWP	*qGWP2a-Z*	umc1065-umc1637	2.05–2.06	CG	3.86	9.18	5.27
				QX	2.56	5.96	2.59
	*qGWP2b-Z*	umc1165-bnlg1017	2.02	CG	2.93	6.66	4.89
	*qGWP6-Z*	bnlg345-umc1424	6.06	QX	3.15	11.85	3.74

Note: CG, Changge, QX, Qixian; LOD, logarithm of odds for each QTL; R^2^, contribution rate; A, additive effect of the QTL. Negative values indicate that the alleles for increased trait value were contributed by the parent Zheng58; positive values indicate that the alleles for increased trait value were contributed by the other parent, Chang7-2.

### Combined QTL detection across four environments

There were 15 QTL associated with EL, ERN, KNR, and GWP across all four environments (Table 6), and they were distributed over chromosomes 2, 3, 4, 5, and 7. The additive effects of the QTL for GWP ranged from −4.06 to 4.45 g, and the QTL *qGWP7-J* showed a positive additive effect of 4.45. The contribution of the additive effect [H^2^ (A)] ranged from 2.28 to 11.27% in the measured traits, and there were two QTL, *qERN*2*-J* and *qERN3b-J*, with higher phenotypic variation values of 11.27 and 9.47%, respectively, for ERN. The interaction of additive effect by environment [H^2^ (AE)] varied from 0.07 to 0.58% of the phenotypic variation. Eight QTL (2 for EL, 2 for ERN, 1 for KNR and 3 for GWP) were detected by combined analysis across the four environments and were also identified in the analyses of the separate environments.

**Table 6:** QTL for ear traits identified by combined analysis among all environments.

Trait	QTL	Interval	Bin	A	H^2^ (A), %	H^2^ (AE), %
EL	*qEL2-J*	umc1261-umc1422	2.02	−0.55	2.80	0.16
	*qEL3-J*	bnlg1904-phi053	3.04	0.45	7.15	0.38
	*qEL4-J*	umc2082-umc2039	4.03	0.63	5.25	0.27
ERN	*qERN2-J*	umc1065-umc1637	2.05–2.06	−0.69	11.27	0.34
	*qERN3a-J*	umc2255-bnlg1904	3.04	−0.22	2.64	0.21
	*qERN3b-J*	umc1528-umc1489	3.07	0.63	9.47	0.07
KNR	*qKNR2-J*	umc1065-umc1637	2.05–2.06	−1.30	6.67	0.15
	*qKNR4-J*	umc2082-umc2039	4.03	0.85	3.73	0.11
	*qKNR5-J*	bnlg1306-bnlg118	5.07	−1.39	7.34	0.16
	*qKNR7a-J*	umc1545-phi057	7.00	0.91	3.51	0.58
	*qKNR7b-J*	umc1799-phi045	7.05	0.47	2.28	0.13
GWP	*qGWP1-J*	bnlg1007-umc1397	1.02	−4.06	2.28	0.18
	*qGWP2a-J*	umc1165-bnlg1017	2.02	−3.35	2.44	0.28
	*qGWP2b-J*	umc1065-umc1637	2.05–2.06	−3.50	3.72	0.52
	*qGWP7-J*	umc1799-phi045	7.05	4.45	2.98	0.22

Note: A, additive effect. Negative values indicate that C7-2 contributed the alleles for increased trait value, and positive values indicate that Z58 contributed the alleles for increased trait value. H^2^ (A)% represents the heritability of the additive effect, and H^2^ (AE)% represents the heritability of the additive × environment interaction effect.

### Analysis of digenic epistatic interactions

Twenty-four pairs of digenic epistatic interactions were identified for six traits with additive × additive (AA) interaction and AA × environment (AAE) interaction effects. The epistatic interactions involved 35 loci distributed on all chromosomes except for chromosome 8 and 10 (Table 7). Only one pair with a significant AAE interaction (P = 0.05) for GWP was identified in CG14 and QX15. Its AAE interaction effect of 0.79% was significantly higher than those of the other traits. This suggests that this epistatic interaction is influenced by the environment. For EL, four significant interactions were detected and found to involve seven loci on chromosomes 2, 5, 6 and 9. The contribution of the AA interactions varied from 1.05 to 3.97%, and the contribution of the AAE interactions varied from 0.19 to 0.63%. Two pairs of loci with significant digenic interactions for ED were detected, and these included 4 loci distributed on chromosomes 1, 2, and 4, with AA interaction effects of 4.26 and 5.87% and AAE interaction effects of 0.14 and 0.27%. A total of six epistatic interactions were identified for ERN, including 11 loci located on chromosomes 2, 3, 4, 6, 7 and 9. Three pairs of epistatic interactions were identified for KNR. Six epistatic interactions were identified for HKW, and their AA interactions explained 3.5, 3.1, 5.3, 4.1, 8.2 and 4.0% of the phenotypic variance. There were three epistatic interactions for GWP, but only one pair of interactions, between bnlg2291-umc1847 and bnlg389-bnlg386, which produced a larger effect than the other interactions.

**Table 7:** Digenic epistatic interactions detected for ear traits in the DH population in four environments.

Trait	QTL_i	Interval_i	Bin	QTL_j	Interval_j	Bin	AA	AAE1	AAE2	AAE3	AAE4	H^2^ (AA), %	H^2^ (AAE), %
EL	2–7	bnlg1175-umc1635	2.09	9–14	bnlg1525-bnlg1588	6.07–6.08	−030***					1.05	0.63
	2–9	umc1065-umc1637	2.05–2.06	9–12	bnlg1270-unc1357	9.05	−0.37***					2.98	0.19
	5–9	bnlg1118-bnlg389	5.07	6–1	bnlg238-phi423796	6.0–6.01	−0.49***					3.97	0.19
	9–4	dupssr6-bnlg244	6.02–6.03	9–14	bnlg1525-bnlg1588	9.07	−0.39***					2.87	0.19
ED	1–12	bnlg1643-bnlg1671	1.08	2–8	umc1635-umc1065	2.05–2.06	0.08***					4.26	0.14
	1–13	bnlg1671-phi265454	1.09	4–8	umc1194-phi092	4.08	−0.13***					5.87	0.27
ERN	3–1	umc2255-bnlg1904	3.04	9–13	unc1357-bnlg1525	9.05	−0.28***					2.59	0.20
	1–3	bnlg1429-bnlg1007	1.02	4–8	unc1194-phi092	4.08	−0.61***					2.18	0.12
	2–6	umc1024-bnlg1175	2.04	9–13	unc1357-bnlg1525	9.05	−0.69***					6.56	0.12
	6–5	umc1014-umc2006	6.04	9–14	bnlg1525-bnlg1588	9.07	0.47***					2.77	0.13
	7–2	phi057-phi034	7.02	9–19	bnlg1129-unc1277	9.08	0.61***					3.32	0.26
	7–8	phi082-phi116	7.05–7.06	9–10	unc1417-phi236654	9.05	0.23***					1.92	0.28
KNR	2–4	umc1422-umc1845	2.02	7–5	umc1112-umc2332	7.03	1.26***					5.30	0.01
	2–4	umc1422-umc1845	2.02	9–4	dupssr6-bnlg244	9.02	−0.49**					1.03	0.28
	7–1	umc1545-phi057	7.02	9–9	unc1494-unc1417	9.05	−0.75***					2.47	0.03
HKW	1–3	bnlg1429-bnlg1007	1.02	4–5	bnlg1621-bnlg2291	4.06	−0.83***					3.47	0.03
	1–3	bnlg1429-bnlg1007	1.02	4–3	umc2039-umc1317	4.03	−0.63***					3.05	0.09
	2–4	umc1422-umc1845	2.02	7–4	bnlg1305-umc1112	7.03	−1.65***					5.28	0.20
	3–4	bnlg197-umc1528	3.06–3.07	9–18	unc2207-bnlg1129	9.08	−1.01***					4.11	0.14
	4–10	bnlg292-umc1173	4.09	7–8	phi082-phi116	7.05	0.49***					8.23	0.35
	4–11	umc1173-umc1109	4.09	7–10	umc1799-phi045	7.06	0.90***					4.00	0.09
GWP	1–14	phi265454-phi064	1.11	2–4	umc1422-umc1845	2.02	3.96***					1.17	0.20
	4–2	umc2082-umc2039	4.03	6–8	bnlg345-umc1424	6.06	4.18***					2.62	0.70
	4–6	bnlg2291-umc1847	4.06	5–10	bnlg389-bnlg386	5.09	−2.28***	2.13***			−3.15***	0.99	0.79

Note: QTL_i and QTL_j – The two QTL involved in the epistatic interaction.

interval_i – The flanking markers of QTL_i.

interval_j – The flanking markers of QTL_j.

AA –additive × additive (AA) interaction effect.

AAE1, AAE2, AAE3 and AAE4 –AA × environment (AAE) interaction effects in CG14, QX14, CG15 and QX15, respectively (non-significant effects are not listed).

H^2^ (AA) – The heritability of AA interaction effects.

H^2^ (AAE) – The heritability of AAE interaction effects.

*, **, *** – 5%, 1%, and 0.5% significance levels.

## Discussion

Previously, an F_2:3_ population, RILs, near isogenic lines (NILs), and DH lines have been used to dissect the genetic basis of quantitative traits in maize^3–5,19–22^. Among the segregating types of populations used for QTL mapping, the F_2:3_ population represents an early generation (transient group), which often affects the accuracy of QTL mapping. Although a RIL is regarded as a permanent population, it generally requires more than 8 generations of continuous selfing, which is a time-consuming and expensive process. By contrast, DH populations can be developed with only two generations in one year; thus, this option is very rapid and inexpensive, making it an ideal population for genetic analysis and QTL mapping^29–32^. In this study, a DH population derived from the elite hybrid ZD958 was used to dissect the genetic basis of grain yield and its components in maize, and a total of 49 QTL and 24 epistatic interactions were detected. These findings could have the potential to help improve grain yield in maize breeding.

Grain yield and its components in maize are complex quantitative traits that are controlled by multiple genes, epistasis and G × E interactions. Over the last 20 years, numerous QTL related to grain yield and its components have been identified by using different segregating populations and association mapping populations. However, many uncertainties and inconsistencies are present in these loci, and these issues might be attributed to several factors, including genetic background (parents, populations and generations), marker types, mapping methods and environments. In this study, a total of 49 QTL were detected in a single environment, whereas 21 QTL for the six investigated traits were detected across two different locations. Although some QTL had lower contributions to the corresponding traits, they did have significant effects on the target traits through interaction with other QTL (epistatic interaction). Our results indicate that the genetic basis of grain yield and its components is controlled by major QTL effects, AA and AAE interaction effects simultaneously. In particular, major QTL with high heritabilities that were detected in different environments simultaneously were considered to have high stability and reliability. Additionally, combined QTL analysis revealed three major QTL hotspots, including pQTL2-1, pQTL2-2 and pQTL6-1, which were located on maize chromosomes 2 and 6 (Supplementary Table 1). At the same time, those chromosome regions were repeatedly identified for several traits of grain yield and its components; for example, the QTL *qEL6b*, which is responsible for EL, and *qKNR6a*, which is responsible for KNR, were located in the same interval (nc012-bnlg345) in multiple environments, indicating that both EL and KNR could be increased simultaneously. Meanwhile, three QTL hotspots in chromosomal bins 2.02 (umc1165-bnlg1017), 2.05–2.06 (umc1065-umc1637), and 6.05 (nc012-bnlg345) were detected both in a single environment and across two different locations. Therefore, these three QTL hotspots related to grain yield may be useful to increase grain yield in maize breeding.

In previous studies, a QTL hotspot responsible for grain-yield-related traits was detected by using a RIL population derived from two inbred lines, Ye478 and Qi319^9,11^; this hotspot was mapped to the bin 2.02 chromosomal region, which contains a QTL detected for ED and GWP in the present study. This genomic region contains some annotated candidate genes, including *ZmLG1*, *ZmMHA*2 and *ZmAST91*, according to MaizeGDB (http://www.maizegdb.org). Among them, the gene *ZmLG1* controls the angle of maize leaves and changes the plant architecture, thereby increasing photosynthetic efficiency and crop yield^33^. *ZmMHA*2 was identified as a functional Fe transporter that promotes Fe uptake and plays an essential role in plant growth and development^34^, while *ZmAST91* could significantly improve crop stress resistance under abiotic stresses^35^. In the bin 2.05–2.06 genomic region, the candidate gene *ZmWri1a* controls the fatty acid content of the mature maize grain and certain amino acids, which can lead to an increase in the weight of the kernel^36^. Another gene, *ZmCDPK*2*4*, which was located in the bin 6.05 genomic region, encodes a calcium-dependent protein kinase and plays a significant role in the regulation of plant growth and development and in responses to various stresses^37^. These colocalized QTL could indicate a single pleiotropic gene, which might act as a regulator to control several traits.

Increasing grain yield is one of the most important targets in maize breeding. Previous studies and production practices have demonstrated that increasing planting density is an effective measure to improve maize grain yield. Planting density is increasing gradually in China and requires new types of hybrids with shorter EL, increased KNR and higher HKW. Of the three major components of grain yield, ERN has the highest heritability and has reliably been used as an important selection target to improve grain yield. In this study, some repetitive or co-located loci for ERN were consistently detected across multiple environments, such as *qERN*2*a* and *qERN*2*-Z*, which were detected in the QTL cluster in bin 2.05–2.06 derived from the elite inbred line C7-2, and a significant QTL for ERN in bin 2.05-2.06 was consistently identified by a combination of meta-QTL analysis and regional association mapping^16^. The elite hybrid ZD958 is based on the most successful heterotic pattern of Reid × Tangsipingtou, which has been widely used in China, and many new varieties have been developed from this hybrid. For example, the famous hybrid Xundan20 (Xun9058 × Xun926) has 2–4 more kernel rows than ZD958. Its male inbred line, Xun926, was modified from the inbred line C7-2. Another commercial hybrid in China, Zhongdan909 (Z58 × HD568) has a parental inbred line, HD568, that was also derived from C7-2, which has 2 kernel rows more than ZD958. These data further confirm that the utilization of inbred line C7-2 to increase ERN is feasible. Therefore, the linked markers of the QTL *qERN*2*a* and *qERN*2*-Z* could be used in marker-assisted selection (MAS) for ERN improvement in maize breeding. Additionally, DH technology has become one of the three core technologies of modern breeding programmes, along with transgenic and MAS breeding technologies^38^. Thus, maize ERN improvement by combining MAS and DH technologies should be highly efficient.

## Materials and methods

### Plant materials

In this study, a population comprised of 161 DH lines was derived from the hybrid ZD958, which is a leading elite maize hybrid developed by Henan Academy of Agricultural Sciences. The DH lines were developed following the procedure described by Prigge *et al*.^39^. Briefly, Zheng58 (Z58) was crossed as the female with Chang7-2 (C7-2) as the male to produce the F_1_ generation (ZD958). The hybrid ZD958 plants were used as the female parent in the field, and the haploid inducer line CAU-5, developed by China Agricultural University^40^, was used as the male parent. Crosses were made by hand pollination, and putative haploids were identified by a colour marker controlled by the *R-nj* gene^41,42^. In the subsequent planting season, haploid seedlings were treated with colchicine and dimethyl sulfoxide (DMSO) to promote haploid genome doubling. After treatment, the haploids were transplanted to the field and selfed to produce DH lines using the methods described by Chen *et al*.^38^. A total of 161 DH lines were obtained for use as experimental materials after reproduction.

### Field experiments

The DH population, ZD958, and its two parents were planted in Qixian (QX14, northern Henan, 35°60′ N lat., 114°20′ E long.) and Changge (CG14, central Henan, 34°1′ N lat., 113°29′ E long.) in the summer of 2014 and then in Qixian (QX15) and Changge (CG15) in the summer of 2015. In each environment, the trial was conducted as a randomized complete block design with two replications. Each experimental plot consisted of a single row with a length of 4 m, a row-to-row distance of 0.66 m, and a plant-to-plant distance within rows of 0.20 m. Two seeds were sown per hill, and the plots were thinned to one seedling per hill at the 5-leave stage. Standard cultivation management practices were used in each environment.

Each plot was harvested by hand at maturity, omitting the two plants at the ends of the plots to avoid border effects. Ten ears from each plot were randomly chosen after natural air-drying to evaluate grain yield and its components, including EL, ED, ERN, KNR, and HKW. The GWP was adjusted to 13% moisture.

### Phenotypic data analysis

The means, standard deviations, correlation coefficients, and kurtosis and skewness of trait distributions for each trait were calculated in SPSS 20.0 software (http://www.spss.com). Variance components were computed using PROC MIXED in SAS^43^ with the following model:

$${Y}_{ijk}=\mu +{G}_{i}+{E}_{j}+G{E}_{ij}+{R}_{k}+{e}_{ijk}$$

where *Y*_*ijk*_ is the performance of the *i*^*th*^ genotype at the *j*^*th*^ environment (location-year combination) in the *k*^*th*^ replication; *μ* is the overall population mean; *G*_*i*_ is the effect of the *i*^*th*^ genotype; *E*_*j*_ is the effect of the *j*^*th*^ environment; *GE*_*ij*_ is the effect of G × E interactions; *R*_*k*_ is the effect of the *k*^*th*^ replication; and *ε*_*ijk*_ is the error. In the model, *G*_*i*_, *GE*_*ij*_ and error effects were considered random effects, and *R*_*k*_ was considered fixed. The broad-sense heritability (*H*^2^) of each trait was estimated as described by Knapp *et al*.^44^. The heritability (*H*^2^) was calculated as:

$${H}^{2}={{\sigma }_{g}}^{2}/({{\sigma }_{g}}^{2}+{{\sigma }_{ge}}^{2}/l+{\sigma }^{2}/lr)$$

where *σ*_*g*_^2^ is the genetic variance, *σ*_*ge*_^2^ reflects G × E interactions, *σ*^2^ is the error variance, *r* is the number of replications, and *l* is the number of environments (location-year combination).

### Genetic map construction

The genomic DNA of the 161 DH families and their parents was extracted from young leaves using the CTAB method^45^. A total of 897 SSR markers distributed over the 10 chromosomes of maize were obtained from MaizeGDB (http://www.maizegdb.org) and used. A total of 119 SSRs with polymorphisms between the two parents were used to construct the genetic map. The PCR products were separated on 6% denaturing polyacrylamide gels with a 19:1 ratio of acrylamide to bisacrylamide and then silver-stained as described by Santos *et al*.^46^. The genetic map was constructed using Mapmaker/EXP V3.0 software^47,48^.

### QTL mapping

QTL mapping in each environment was conducted by the composite interval mapping method (CIM) using the software Windows QTL Cartographer version 2.5^49^. For CIM, mixed-model-based composite interval mapping was undertaken by using a forward-backward stepwise procedure with a threshold of P = 0.05 to select cofactors, and the window size was set to 10 cM. The threshold for declaring the presence of a significant QTL for each trait was determined after 1000 permutations at a significance level of P = 0.05. The confidence interval calculated by the odds ratio reduced by a factor of 10 was averaged for each QTL^50^.

Combined QTL analysis, the digenic epistatic effects of QTL, the heritability of additive effects, AAE effects, epistatic effects, and the prediction of superior genotypes based on the datasets of all experimental environments were performed by QTLNetwork software version 2.1^51^. The total heritability (*H*^2^_*T*_) was estimated by the following equation:

$${H}_{T}^{2}={H}_{G}^{2}+{H}_{GE}^{2}=({H}_{A}^{2}+{H}_{D}^{2}+{H}_{I}^{2})+({H}_{AE}^{2}+{H}_{DE}^{2}+{H}_{IE}^{2})$$

where *H*^*2*^_*T*_ is the total heritability; *H*^*2*^_*G*_ is the heritability of genetic effects, which included *H*^*2*^_*A*_ (heritability of additive effects), *H*^*2*^_*D*_ (heritability for dominance effects) and *H*^*2*^_*I*_ (heritability of epistasis effects including AA, additive × dominant (AD), and dominant × dominant (DD); *H*^*2*^_*GE*_ is the heritability of G × E interaction effects, which included *H*^*2*^_*AE*_ (heritability of additive × environment interaction effects), *H*^*2*^_*DE*_ (heritability of dominance × environment interaction effects) and *H*^*2*^_*IE*_ (heritability of epistasis-environment interaction effects including AAE, AD × environment, and DD × environment). The testing window, walking speed (the distance between two adjacent test points in a genome scan) and filtration window were set at 10, 1 and 10 cM, respectively. The experiment-wise type I error for candidate interval selection and putative QTL detection and the significance level for QTL effects were set at P = 0.05. The F-statistic based on Henderson Method III was used to determine significance, and 1000 permutations were used to control the genome-wide false positive rate^52^.

QTL denotation followed the rules suggested by McCouch *et al*.^53^: the name of each QTL was defined starting with a lowercase ‘*q*’, then the trait name in capital letters, followed by the chromosome number where the QTL was detected. For example, *qEL*5 refers to a QTL for EL detected on chromosome 5. If there was more than one QTL for one trait on the same chromosome, lowercase letters were added after the chromosome number to distinguish them; for example, *qKNR*6*a* and *qKNR*6*b* were, respectively, the first and second QTL for KNR on chromosome 6. In addition, the four environments were divided into two planting zones (QX14 and QX15 are in the northern part of Henan Province, and CG14 and CG15 are in the central part of Henan Province) according to environmental differences. QTL were identified by separate analysis for these two zones. ‘-Z’ and ‘-J’ were added after the names of the corresponding QTL to distinguish them from single-environment QTL.

## Conclusions

In total, 49 QTL were detected for six traits related to grain yield across four different environments at two locations over 2 years using a DH population, and 8, 6, 12, 8, 8 and 7 QTL were detected for EL, ED, ERN, KNR, HKW and GWP, respectively. The phenotypic contribution percentage of each QTL ranged from 5.71 to 17.84%. One QTL (*qERN2a*) was consistently detected in four environments, and its contribution varied from 8.5% to 11.5%. The QTL *qEL4*, *qEL6b*, *qED2a*, *qERN2a*, *qKNR2a*, *qKNR6a*, *qHKW5a*, and *qGWP2a* were also detected in two or three environments simultaneously. In addition, 15 significant QTL in the combined analysis and 21 QTL across both planting locations were related to EL, KNR, ERN, and GWP; no QTL were identified for ED or HKW. There were 24 pairs of epistatic interactions for the six measured traits. Importantly, three obvious QTL hotspots associated with yield components were found in maize chromosomal bins 2.02, 2.05–2.06, and 6.05. This study will not only contribute to a theoretical basis for predicting potentially superior yield traits in maize but also support MAS in maize breeding programmes.

## Supplementary information

Supplementary Information.
