# Publisher Correction: Mapping of QTL for Grain Yield Components Based on a DH Population in Maize

**DOI:** 10.1038/s41598-020-70123-w

**Published:** 2020-07-31

**Authors:** Jiwei Yang, Zonghua Liu, Qiong Chen, Yanzhi Qu, Jihua Tang, Thomas Lübberstedt, Haochuan Li

**Affiliations:** 1 Agronomy College of Henan Agricultural University/Key Laboratory of Wheat and Maize Crops Science/Collaborative Innovation Centre of Henan Grain Crops, Zhengzhou, 450002 China; 2 Department of Agronomy, Iowa State University, Ames, IA 50011 USA

Correction to: *Scientific Reports*10.1038/s41598-020-63960-2, published online 27 April 2020

This Article contains errors in Figure 1.

Firstly, the QTL EL14CG, denoted as the double line circle, is missing from chromosome 6 between the molecular markers phi031 and umc1014.

Secondly, in the key for the QTL 100-kernal weight,

- “HGW 14CG”
- “HGW 14QX”
- “HGW 15CG”
- “HGW 15QX”

should read:

- “HKW 14CG”
- “HKW 14QX”
- “HKW 15CG”
- “HKW 15QX”

The correct Figure 1 appears below.

**Figure 1:**
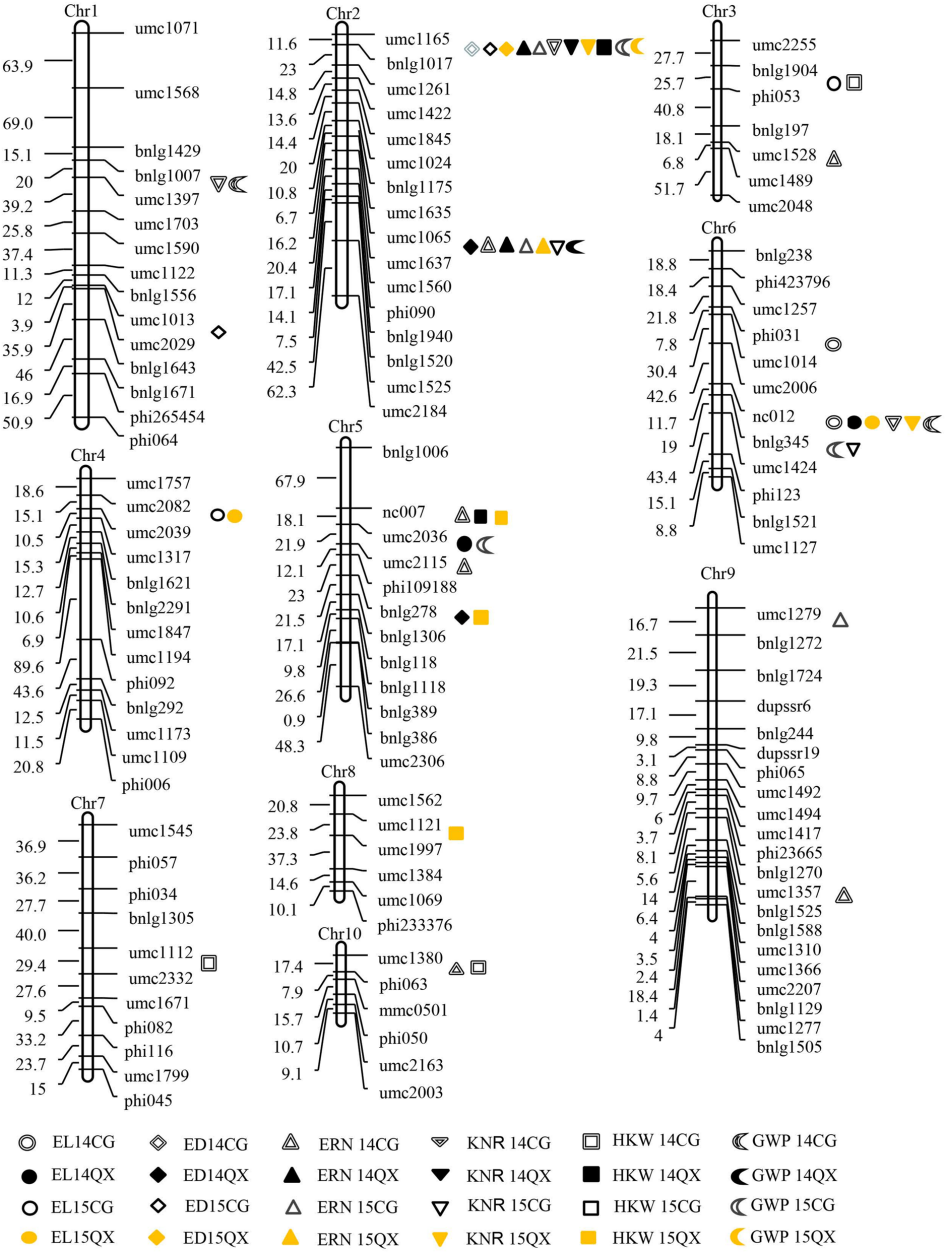
.

