# Supplementary material for: Mapping of QTL for Grain Yield Components Based on a DH Population in Maize
Source: Sci Rep. 2020 Apr 27;10:7086. doi: 10.1038/s41598-020-63960-2 (PMC7184729; doi:10.1038/s41598-020-63960-2)
Supplement: Supplementary file 1 — Supplementary Information. [file 41598_2020_63960_MOESM1_ESM.pdf]

Mapping of QTL for Grain Yield Components Based on a DH Population in Maize

Jiwei Yang[1*], Zonghua Liu[1*], Qiong Chen[1], Yanzhi Qu[1], Jihua Tang[1], Thomas Lübberstedt[2], Haochuan Li[1+]

[1]Agronomy College of Henan Agricultural University/ Key Laboratory of Wheat and Maize Crops Science/ Collaborative Innovation Centre of Henan Grain Crops, Zhengzhou 450002, China

[2]Department of Agronomy, Iowa State University, Ames IA 50011, USA

[+] Corresponding author:

Haochuan Li

Tel.: +86-371-56990188

E-mail: lihaochuan1220@163.com

[*]Jiwei Yang and Zonghua Liu contributed equally to this work

SUPPLEMENTARY INFORMATION

This file includes one supplementary table (Supplementary Table S1) and two supplementary figures (Supplementary Figure S1 and S2).

Supplementary Table S1. QTL identified for more than one trait across four environments.

Note: [a] The name of each pleiotropic QTL; [b] chromosome; [c] position indicates the physical position; [d] Flanking markers, the markers to the each side of the QTL.

Supplementary Figure S1. Frequency distribution of DH families for maize ear traits in four environments over 2014 and 2015 years.

Note: CG14 and QX14 represent Changge and Qixian in 2014; CG15 and QX15 represent Changge and Qixian in 2015. EL(ear length), ED(ear diameter), ERN(ear row number), KNR(kernel number per row), HKW(100 kernels weight), GWP(grain weight per plant).

Supplementary Figure S2. Molecular linkage map of DH families and distribution of QTL for ear traits at two different locations over 2 years.

Note: the markers denoted peak positions of QTL. CG and QX represent Changge and Qixian, respectively; EL (ear length), ED (ear diameter), ERN (ear row number), KNR (kernel number per row), HKW (100-kernel weight), GWP (grain weight per plant). Numbers on the left side of each chromosome are the genetic distances between two flanking markers with the unit of centiMorgan (cM). Marker names are shown on the right of each chromosome.

Supplementary Table S1. QTL identified for more than one trait across four environments.

pQTL[a]	Chr.[b]	Pos.[c]	Marker interval[d]	No. of QTL	Trait
pQTL1	1	156.0-160.0	bnlg1007-umc1397	2	KNR/GWP
PQTL2-1	2	0-8.0	umc1165-bnlg1017	11	ED/ERN/KNR/GWP/HKW
PQTL2-2	2	114.9-131.1	umc1065-umc1637	7	ED/ERN/KNR/GWP
PQTL3-1	3	31.7-53.4	bnlg1904-phi053	2	EL/HKW
pQTL5-1	5	69.9-75.9	nc007-phi109188	3	ERN/HKW
pQTL5-2	5	147-164.5	bnlg278-bnlg1306	2	ED/HKW
pQTL6-1	6	125.2-135.2	umc2006-nc012	2	ED/GWP
pQTL6-2	6	139.8-147.8	nc012-bnlg345	8	EL/KNR/GWP
pQTL6-3	6	182.5-200.5	umc1424-phi123	3	GWP/HKW
pQTL10	10	2.0-12.0	umc1380-phi063	2	ERN/HKW

Note: [a] The name of each pleiotropic QTL; [b] chromosome; [c] position indicates the physical position; [d] Flanking markers, the markers to the each side of the QTL.

Supplementary Figure S1. Frequency distribution of DH families for maize ear traits in four environments over 2014 and 2015 years.

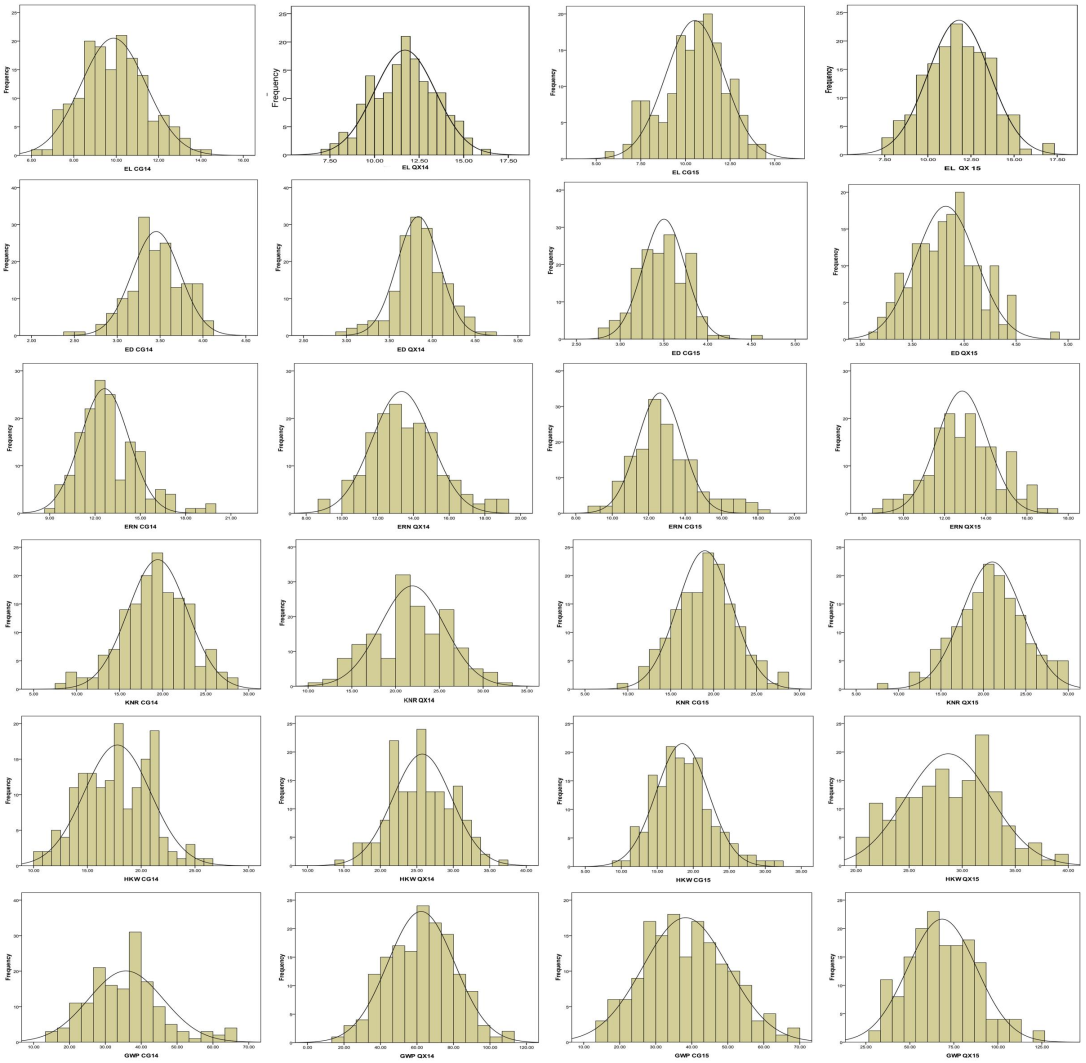

Note: CG14 and QX14 represent Changge and Qixian in 2014; CG15 and QX15 represent Changge and Qixian in 2015. EL(ear length), ED(ear diameter), ERN(ear row number), KNR(kernel number per row), HKW(100 kernels weight), GWP(grain weight per plant).

Supplementary Figure S2. Molecular linkage map of DH families and distribution of QTL for ear traits at two different locations over 2 years.

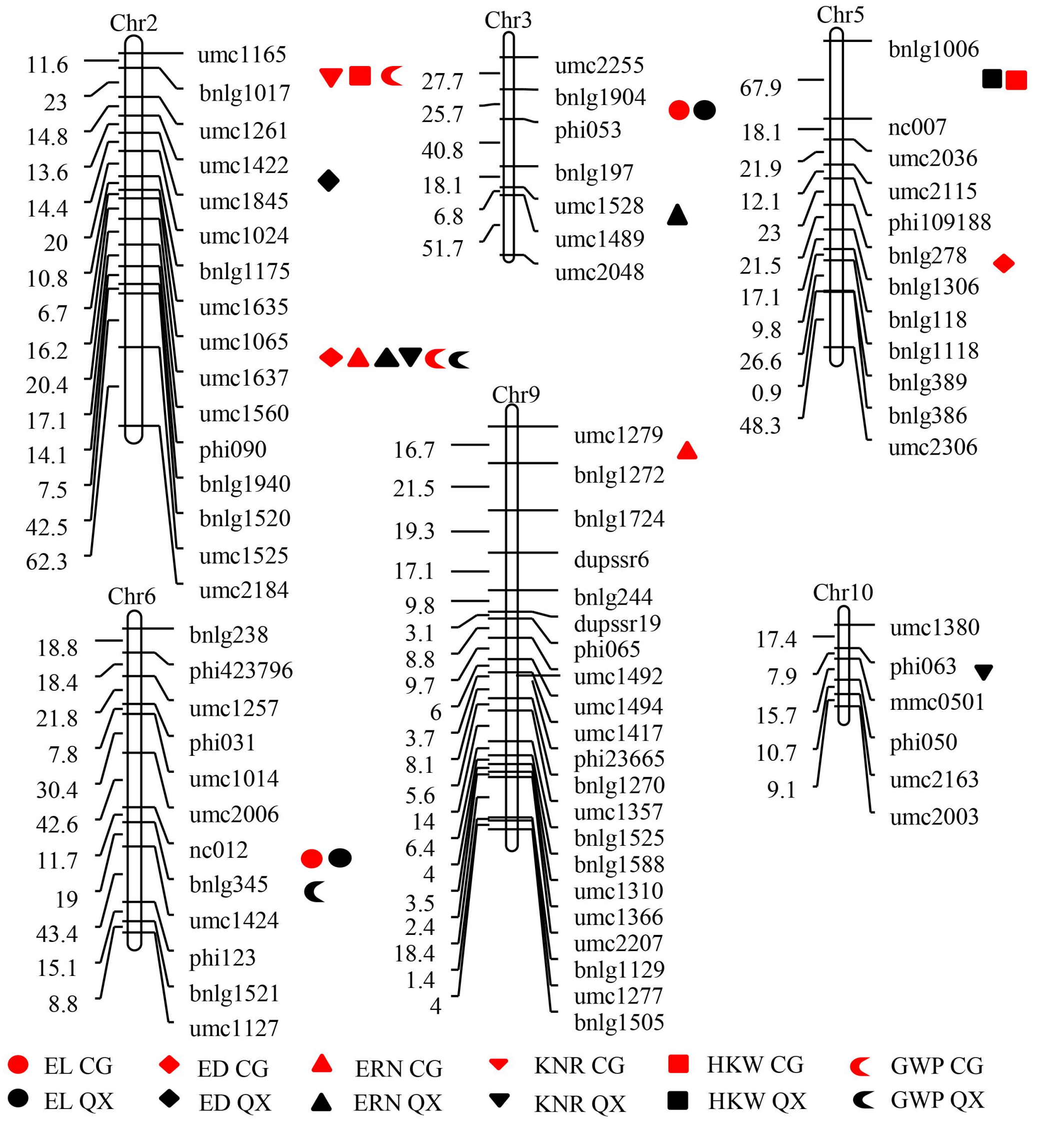

Note: the markers denoted peak positions of QTL. CG and QX represent Changge and Qixian, respectively; EL (ear length), ED (ear diameter), ERN (ear row number), KNR (kernel number per row), HKW (100-kernel weight), GWP (grain weight per plant). Numbers on the left side of each chromosome are the genetic distances between two flanking markers with the unit of centiMorgan (cM). Marker names are shown on the right of each chromosome.
